# Complete genome sequence of *Dehalococcoides mccartyi* strain Y2, a tetrachloroethene-dechlorinating anaerobe isolated from river sediment

**DOI:** 10.1128/mra.00254-26

**Published:** 2026-06-22

**Authors:** Mengdie Qi, Xiuying Li, Huijuan Jin, Yiru Cui, Xiaocui Li, Jun Yan, Jingjing Wang

**Affiliations:** 1 Key Laboratory of Pollution Ecology and Environmental Engineering, Institute of Applied Ecology, Chinese Academy of Sciences 74763, Shenyang, Liaoning, China; 2 Shenyang Agricultural University 98428, Shenyang, Liaoning, China; 3 Provincial University Key Laboratory for Risk Prevention and Control of Emerging Contaminants, Nanjing Normal University 12534, Nanjing, Jiangsu, China; 4 School of Environment, Nanjing Normal University 12534, Nanjing, Jiangsu, China; Rochester Institute of Technology, Rochester, New York, USA

**Keywords:** *Dehalococcoides*, reductive dehalogenate, tetrachloroethene, heavy-metal stress

## Abstract

*Dehalococcoides mccartyi* strain Y2, an anaerobe isolated from river sediment, reductively dechlorinates tetrachloroethene to *cis*-1,2-dichloroethene and *trans*-1,2-dichloroethene. The complete genome of strain Y2 comprises a 1,401,283 bp circular chromosome with a G + C content of 47.95% and contains 24 reductive dehalogenase genes.

## ANNOUNCEMENT

Co-contamination of chlorinated solvents, such as tetrachloroethene (PCE), and heavy metals is common at industrial sites (1, 2). Among these, zinc ions are particularly inhibitory to reductive dechlorination (3, 4). *Dehalococcoides mccartyi* (*Dhc*) strains are key players for bioremediation of chlorinated solvents in contaminated subsurface environments (5); however, their ability to tolerate heavy-metal stress remains poorly understood.

A *Dhc* enrichment culture was established by inoculating freshwater sediment collected from the Xi River (Shenyang, Liaoning, China) into bicarbonate-buffered (pH 7.2–7.3), reduced basal medium (6) supplemented with 50 mg/L Zn^2+^. Cultures were incubated at 30°C and amended with 0.5 mM aqueous-phase PCE as the electron acceptor, 5 mM acetate as the carbon source, and 10 mL H_2_ (i.e., 408 μmol) as the electron donor. Transfers were performed approximately monthly after complete conversion of PCE to *cis*-1,2-dichloroethene and *trans*-1,2-dichloroethene. A *Dhc* isolate, designated strain Y2, was obtained in 2023 using a dilution-to-extinction protocol (7). Culture purity was verified by PCR amplification of the 16S rRNA gene using universal bacterial primers 27F and 1492R (8), followed by Sanger sequencing. Strain Y2 shared 98.73% 16S rRNA gene sequence identity with *Dhc* strain 195^T^ (accession no. AF004928). Biomass was harvested from 1.5 L strain Y2 culture by centrifugation at 15,480 × *g* for 10 min. Genomic DNA was extracted using the PureLink Genomic DNA Extraction Kit (Thermo Fisher Scientific, Waltham, MA, USA). Whole-genome sequencing was performed using Illumina NovaSeq PE150 and PacBio Sequel II platforms. For Illumina sequencing, a paired-end library was prepared using the TruSeq Nano DNA Sample Prep Kit (Illumina, San Diego, CA, USA), generating 8,425,236 raw reads (2 × 150 bp) with 95.83% of bases at Q30 or higher. For PacBio sequencing, high-molecular-weight DNA was processed using the BluePippin (Sage Science, Beverly, MA, USA), and SMRTbell libraries were constructed using the PacBio Express Template Prep Kit 2.0 (Pacific Biosciences, Menlo Park, CA, USA), generating 423,976 long reads (*N*_50_ = 1,635 bp). Default parameters were used unless otherwise stated. Raw reads were trimmed and quality controlled using Trimmomatic v0.36 with SLIDINGWINDOW:4:15 and MINLEN:75 (9). Genome assembly was performed using a Unicycler-mediated hybrid workflow, in which Illumina short reads were first assembled with SPAdes v3.9.0, and PacBio long reads were used for contig bridging, repeat resolution, and circularization (10, 11).

The strain Y2 genome consists of a single circular chromosome, and its genomic features are summarized in Table 1. Annotation with the NCBI Prokaryotic Genome Annotation Pipeline v6.9 identified the predicted genes and RNA features (11). ANI analysis showed 86.33% identity between strain Y2 and strain 195^T^, indicating substantial genomic divergence. Following the current taxonomic convention, we retained the designation *Dehalococcoides mccartyi* strain Y2 (5). Strain Y2 harbors 24 non-identical reductive dehalogenase catalytic subunit A (*rdhA*) genes, exceeding the 17 intact reductive dehalogenase homologous genes reported in strain 195^T^ and suggesting a relatively broad organohalide-respiration potential. Several putative heavy-metal resistance components were also identified, including a heavy-metal-translocating P-type ATPase (12, 13), two metal efflux pump genes (*mntP* and *arsB*), and an *arsR/smtB* family transcriptional regulator (14). These features suggest that strain Y2 may persist and sustain reductive dechlorination under heavy-metal stress.

**TABLE 1:** Genomic features of *Dehalococcoides mccartyi* strain Y2

Features	Value
Genome size	1,401,283 bp
GC content (%)	47.95
Number of chromosomes	1
Contig number	1
*N*_50_	1.4 Mb
*L*_50_	1
PacBio long-read coverage	12×
Completeness (%)	98.9
Contamination (%)	0.25
Total genes	1,504
CDS	1,447
Number of RNAs	52
Number of rRNAs	3
Number of identified genes	1,504
Number of identified CDSs	1,447
Number of identified *rdhAs*	24
Heavy-metal resistance genes	P-type ATPase, *mntP, arsB, arsR/smtB*

## Data Availability

The strain Y2 genome sequence has been deposited in the GenBank under the accession number CM137044. The BioSample and BioProject numbers are SAMN54473342 and PRJNA1399081, respectively. The raw sequencing reads have been submitted to the Sequence Read Archive (SRA) under the accession number SRR36718677.
